# Subliminal unconscious conflict alpha power inhibits supraliminal conscious symptom experience

**DOI:** 10.3389/fnhum.2013.00544

**Published:** 2013-09-05

**Authors:** Howard Shevrin, Michael Snodgrass, Linda A. W. Brakel, Ramesh Kushwaha, Natalia L. Kalaida, Ariane Bazan

**Affiliations:** ^1^Program of Research on Unconscious Processes, Ormond and Hazel Hunt Laboratory, Department of Psychiatry, University of Michigan, Ann Arbor, MI, USA; ^2^Faculté des Sciences Psychologiques et de l'Education, Service de Psychologie Clinique et Différentielle, Université Libre de Bruxelles (ULB)Brussels, Belgium

**Keywords:** anxiety, defense mechanisms, repression, alpha, EEG/ERP, avoidance, subliminal, neuropsychoanalysis

## Abstract

Our approach is based on a tri-partite method of integrating psychodynamic hypotheses, cognitive subliminal processes, and psychophysiological alpha power measures. We present ten social phobic subjects with three individually selected groups of words representing unconscious conflict, conscious symptom experience, and Osgood Semantic negative valence words used as a control word group. The unconscious conflict and conscious symptom words, presented subliminally and supraliminally, act as primes preceding the conscious symptom and control words presented as supraliminal targets. With alpha power as a marker of inhibitory brain activity, we show that unconscious conflict primes, only when presented subliminally, have a unique inhibitory effect on conscious symptom targets. This effect is absent when the unconscious conflict primes are presented supraliminally, or when the target is the control words. Unconscious conflict prime effects were found to correlate with a measure of repressiveness in a similar previous study (Shevrin et al., 1992, 1996). Conscious symptom primes have no inhibitory effect when presented subliminally. Inhibitory effects with conscious symptom primes are present, but only when the primes are supraliminal, and they did not correlate with repressiveness in a previous study (Shevrin et al., 1992, 1996). We conclude that while the inhibition following supraliminal conscious symptom primes is due to conscious threat bias, the inhibition following subliminal unconscious conflict primes provides a neurological blueprint for dynamic repression: it is only activated subliminally by an individual's unconscious conflict and has an inhibitory effect specific only to the conscious symptom. These novel findings constitute neuroscientific evidence for the psychoanalytic concepts of unconscious conflict and repression, while extending neuroscience theory and methods into the realm of personal, psychological meaning.

## Introduction

Oddly as psychoanalysis seems to diminish in the eyes of many, neuroscience appears to be taking a long second look at psychoanalysis. Interest and acceptance of an unconscious has been growing. Leading theoreticians have begun grappling with Freud's theory, even thinking of it as the most comprehensive available to psychiatry (Kandel, 1998, 1999), and offering the best account of recent neuroscience findings (Carhart-Harris and Friston, 2010). A growing number of psychoanalysts are going back to Freud's neuroscience roots, finding in his posthumous Project (1950) insightful anticipation of modern neuroscience concepts (Pribram and Gill, 1976). Some have suggested that neuroscience provides the basic science testing ground to avoid the circularity of many psychoanalytic explanations (Rubinstein, 1977). Yet there remain powerful obstacles on both sides to a true fruitful scientific exchange. The neuroscience investigation of the neural correlates of unconscious processes is often limited to automatic biases, ignoring the importance of unconscious conflict, the role of personal meaning, and unique unconscious processes like repression. In contrast, we present the unconscious that is subject to individual meaning (contained in the unconscious conflict unique to each individual), is comprised of complex unconscious emotional processing including repression, and plays a causative role in the manifestation of symptoms as in social phobias. Moreover, we show that these processes are instantiated in identifiable brain events.

Perhaps the two most controversial psychoanalytic clinical concepts are unconscious conflict and repression. On the basis of these two concepts psychoanalysts seek to explain a variety of psychiatric symptoms and how they can be successfully treated. Unconscious conflict is presumed to arise from opposing desires, working largely unconsciously and subject to unconscious efforts at inhibiting or repressing the conflict that can create great anxiety, shape how a person responds to the challenges of life, and can create a variety of psychiatric symptoms such as the social and specific phobias we have investigated.

Yet unconscious conflict theory is not the only theory of psychopathology psychoanalysts espouse. Psychoanalysts often refer to “conflict theory” as “classical” both in complimentary and pejorative ways. Adherents see it as the original Freudian theory; critics see it as essentially outmoded and replaceable by newer conceptualizations. These different theories all suffer from one fatal flaw-none have achieved the empirical support scientifically required to pass muster as an established scientific theory. Whether you select one theory or another is more a matter of preference than one based on defensible evidence (Rubinstein, 1977). We decided to exercise our preference for conflict theory mainly because it was historically the first and has lasted a considerable time; it was also the one we were best acquainted with by training and clinical experience.

Without doubt one of the most trenchant critics of Freud, Grunbaum (1984) has pointed out that empirical support must come from methods that can be shown to be independent of the clinical method, otherwise circularity is the ever present danger. In response to these challenges, we applied our research method in order to find independent evidence for the validity of the constructs of repression and unconscious conflict. At the same time we believed our research method is theory neutral and could be used by other theories to test their basic hypotheses.

Within psychoanalysis sharply critical voices along the same lines as Dr. Grunbaum have been heard as well. Outstanding theoreticians such as Rapaport (1959) have written extensively on these same issues. It is important to note that Rapaport's influence as a thinker and theoretician went beyond psychoanalysis. He was an early influence on Daniel Kahneman, as Kahneman (2003) noted in his Autobiography. Kahneman's monograph “Attention and Effort” contained a theory of attention as a limited resource to which Rapaport had originally led him (Kahneman, 1973, 2003). Kahneman also incorporated the concept of psychic energy, another important Rapaport theoretical preoccupation and central to Freud's concept of repression that Kahneman found useful and renamed effort (Kahneman, 1973, 2003). Kahneman was intending to spend more time studying with Rapaport; unfortunately Rapaport died prematurely at 50 before Kahneman could pay his return visit. Interactive contacts between psychoanalysts and cognitive and neuroscientists, as between Rapaport and Kahneman, have likely occurred a number of times, but were not sustained or further exploited.

Our research group set about some years ago to venture onto this difficult terrain. The tripartite approach was a natural next step from the senior author's previous research in which he was the first to report event related potential markers of subliminal unconscious processes (Shevrin and Fritzler, 1968), to demonstrate unconscious inhibition (Snodgrass et al., 1993a,b), and to devise methods for investigating two different modes of thought related to Freud's concepts of primary and secondary process: [Shevrin and Luborsky (1961), Brakel et al. (2000), see also Brakel and Shevrin (2003) for a comparison of Freud's theory of primary and secondary processes with more recent cognitive dual process theories].

Our early studies were designed as straightforward cognitive investigations incorporating subliminal and electrophysiological methods. What they all lacked was any clinical data from which the existence of unconscious processes and repression were inferred. The new method sought to correct this lack by including in depth interviews of patients who were also the research participants on whom the inferences derived from the interviews were tested. The initial study we conducted utilizing this tripartite method involved social phobic subjects (described in section The Initial Social Phobia Study: Establishing Clinical and Brain Evidence for Unconscious Conflict). A detailed account of the tripartite method and the encouraging findings emerging from this initial study were published in Consciousness and Cognition (Shevrin et al., 1992) and subsequently in a book length treatment including additional findings and three detailed case studies (Shevrin et al., 1996). The main objective of the initial study was to determine that our method would provide objective evidence for the existence of unconscious conflict; secondarily it was hoped that the data would also show a repressive cause and effect relationship between unconscious conflict and conscious symptom experience. This first study (summarized below in section The Initial Social Phobia Study: Establishing Clinical and Brain Evidence for Unconscious Conflict) succeeded with its primary objective, but results for the second were ambiguous at best. We realized that we lacked a neural correlate for the nature of the causal relationship between unconscious conflict and conscious symptom experience. A series of subsequent studies (summarized below Previous Studies With Spider Phobia: Alpha Power Serves as an Inhibiting Brain Mechanism in Phobic Experience) were aimed at investigating the role of alpha power as an inhibitory agent that might provide the neural causal link. Following the presentation of these two sets of earlier findings, we describe our new previously unpublished study (section The New Investigation: Establishing a Repressive Causal link Between Unconscious Conflict and Conscious Symptom Experience) replicating and extending these earlier findings, and we believe identifying the neural correlate we were seeking.

## The initial social phobia study: establishing clinical and brain evidence for unconscious conflict

The subjects were eleven social phobics who met DSM IV-R criteria. Four clinician judges selected individual words to be presented as stimuli for each subject. In a within subject design three groups of words were selected. The words were chosen individually for each subject to represent the unconscious conflict, conscious symptom experience, and a group of general negative valence words [see Shevrin et al.(1996, p. 139 Appendix B) for a detailed description of the word selection procedure]. The three word groups were equated for frequency, length, and part of speech (nouns and verbs). All words were presented subliminally and supraliminally. The brain responses were measured by time-frequency features derived from event-related potentials (ERPs). The essential idea behind time-frequency analytic approaches is simple: While standard ERP methods depict brain responses in two dimensions (time and amplitude), time-frequency methods add a third dimension—frequency. Time-frequency methods thus represent event-related brain responses as the frequency and amplitude (more accurately, power) present at each time bin on each trial, rather than only the amplitude at each time bin, as in usual ERP methods. “Time-frequency features,” then, refer to points in this three-dimensional space. The application of time-frequency features was a relatively new approach to ERPs at the time pioneered by co-author Williams (Moser and Avnon, 1986; Williams et al., 1987, 1994; Cohen, 1989; Williams and Jeong, 1989, 1992; Zaveri et al., 1992). See Supplement 1 and Shevrin et al. (1992) for further description.

In this earlier study, we used discriminant analysis to select the t-f features which best differentiated the critical unconscious conflict and conscious symptom word stimulus categories (vs. general negative words). These disciminant analysis/classification findings were then internally cross-validated by performing a standard development vs. test set (i.e., odd vs. even-numbered trials) analysis. The t-f feature analysis and its use are explained in the book length presentation (Shevrin et al. 1996, p. 139 Appendix B).

The main finding was a significant two way interaction between word category (unconscious conflict vs. conscious symptom) and duration (subliminal vs. supraliminal). The brain responses to the unconscious conflict words were more correctly classified subliminally as compared to the general negative valence control; the reverse was found for the brain responses of the conscious symptom words (Shevrin et al., 1992, 1996). A second finding concerned repressiveness as measured by a Hysteroid-Obsessoid Questionnaire (HOQ) that the subjects completed. The HOQ is a self-report personality trait instrument originally developed to measure obsessive vs. hysterical personality styles (Caine and Hawkins, 1963; Caine and Hope, 1967). Psychoanalytic theory suggests repressive defenses should be prominent in those with the latter style. Further, earlier work (Ludolph, 1981; Shevrin et al., 2002a) found significant positive relationships between hysterical/repressive HOQ scores and various other indicators of repressive defenses (e.g., related indexes on the Rorschach). These two tests arrive at measures through largely different methods. Here, in the initial social phobic study, the HOQ predicted better classification of UC stimuli subliminally, but poorer classification of UC stimuli supraliminally. This finding suggested that an inhibitory repressive process was at work when the unconscious conflict words were presented supraliminally, inhibiting conscious recognition of their unconscious significance. The comparable correlations for the conscious symptom words and the control words were both non-significant (Shevrin et al., 1992, 1996). These two findings, inferred from clinical material by subjective clinical judgments, and hypothesized to be a cause of the symptom, were paralleled by objective time-frequency measures of unconscious processes.

The conflict stimuli selected were unique for each subject, a practice rare in cognitive research. Nevertheless, the positive results demonstrated that these different stimuli across subjects produced similar results (Shevrin et al., 1992, 1996). We further tested whether the unconscious conflict and conscious symptom words formed unique categories by scrambling the words across categories to form new pseudo-categories. We used an information flow measure (Kushwaha et al., 1992), an adaptation of Shannon-type information measures, to assess stimulus-related information flow between pairs of electrodes. Although this measure cannot tell us what aspects of the ERP response (e.g., amplitude, frequency, etc.) are carrying this information, it does—critically—measure specifically stimulus category-related information (vs., e.g., other information flow analytic techniques which do not distinguish category-related vs. background activity). See Supplement 1 of the current manuscript for further explanation the information flow technique, and Kushwaha et al. (1992) for full details. Substantively, Kushwaha et al. found that significantly more stimulus related information flowed between electrodes for the true categories than for the pseudo-categories, convergently indicating that the former were indeed true categories. Additionally, we found greater information flow when the unconscious conflict words were presented subliminally as compared to supraliminally (Kushwaha et al., 1992).

The evidence from the first study with social phobics established the existence of unconscious conflict on the basis of clinical and independent non-clinical methods. It seemed clear that the unconscious conflict stimuli formed unique, individually meaningful categories. However, what was not clear is how these stimuli acted as causes of conscious symptom experience.

## Previous studies with spider phobia: alpha power serves as an inhibiting brain mechanism in phobic experience

We then set about to investigate the link between unconscious conflict and conscious symptom experience. In our subsequent studies, we shifted our primary measure of physiological brain activity from time-frequency features to alpha power measures. We did so because much recent evidence had suggested that alpha power may be an important brain mechanism for inhibiting task irrelevant stimuli. For example, if the same frequency light was presented to each eye and the subject was instructed to pay attention only to the left eye, alpha power contralateral to the right eye increased significantly. Alpha power played a role in inhibiting attention to the right eye (Kelly et al., 2006). If this inhibitory function were generalizable it might provide the inhibitory function involved in repression. Charles Brenner, a leading psychoanalytic theoretician, had earlier hypothesized that psychodynamic defenses like repression were made up of normal cognitive functions that were put to specific unconscious motivational uses (Arlow and Brenner, 1964; Brenner, 1976, 1982). It would follow from this hypothesis that alpha power might provide the inhibitory function needed for repression, while the motivation for the inhibition would derive from the person's unconscious conflict. If so then alpha power might be the means by which to quantitatively measure the causal inhibitory link between unconscious conflict and conscious symptom experience.

We set about conducting a series of experiments with spider phobics. We presented phobic spider images and controls, subliminally and supraliminally. An FFT measured alpha power in the 8–13 frequency range. Subjects also completed a standard detection procedure with subliminal images, and rated their fear of spiders before and after the subliminal presentations (Shevrin et al., 2010; see Supplement 2 for the published poster abstract). Snake phobics served as a control for phobic state and rectangles served as a neutral stimulus control. In this study with spider phobics we did not assess unconscious conflict; our limited aim was to see if for spider phobics alpha power inhibited responses to spider stimuli and other spider related responses. If this turned out to be the case, then the inhibiting function of alpha power could be generalized beyond the purely cognitive inhibition of distracting or irrelevant stimuli to inhibiting relevant but emotionally disturbing stimuli. It might then approximate, from a psychoanalytic standpoint, a defense resembling repression once it could be linked to a related unconscious conflict.

The results were encouraging. Increased alpha power correlated with: (1) diminished attention to the phobic spider stimulus as reflected in smaller N100 amplitude and delayed N100 latency, (2) below chance (inhibited) detection of spider stimuli in a standard detection procedure, (3) greater self-reported levels of spider fear, (4) worsening spider fear after repeated subliminal exposure. Control results for snake phobics and rectangles for these comparisons were all non-significant (Shevrin et al., 2010; see Supplement 2 for the published poster abstract).

The results from this study with spider phobics demonstrated that alpha power went far beyond inhibiting attention to purely cognitively irrelevant stimuli. Rather, alpha power played an inhibitory role: (1) when the perceptual stimulus was emotionally significant (a feared spider), (2) when the task was to attend to an emotionally significant stimulus, (3) when the phobic stimulus elicited greater anxiety and fear, and (4) when multiple subliminal exposures did not decrease phobic spider fear. The finding that avoidance worked against improvement following multiple subliminal presentations of spiders suggested that the research might have treatment implications (Shevrin et al., 2010). It was as if the inhibitory process was flexible and not bound to a particular task. Rather, it served a specific unconscious motivation that could be imagined in these words, “I am afraid of spiders and I don't want to have anything to do with them. I will inhibit any process that increases the likelihood of exposure to spiders.” When put in these terms it begins to sound like repression. Indeed, as cited previously, Brenner defined defenses from a psychoanalytic standpoint as a particular cognitive function linked to a particular motive. In this instance the particular cognitive function was inhibition associated with alpha power, and the motive was to avoid or minimize fear or anxiety with respect to spiders. These strong indications that alpha power may serve as the inhibitory brain mechanism of repression lead us to focus on the role of alpha power in the new, original study to be reported below.

## The new investigation: establishing a repressive causal link between unconscious conflict and conscious symptom experience

Much cognitive research had long called attention to the central role of avoidance in anxiety disorders. In our research we sought to demonstrate that avoidance was related to unconscious conflict and repression, and generalizable across many different tasks. Moreover, these processes could all go on unconsciously. It is notable that recently, Klimesch et al. (2011) have provided extensive cognitive neuroscience evidence that alpha power serves a general inhibitory function not limited, as heretofore believed, to distracting and irrelevant stimuli. Its inhibitory function can be applied to different tasks and stimuli determined by a variety of top down influences such as expectations. These considerations parallel our own conclusions reported in Shevrin et al. (2010).

In our first social phobia study (section The Initial Social Phobia Study: Establishing Clinical and Brain Evidence for Unconscious Conflict) we showed that unconscious conflict based on in-depth clinical data exists, but we could not claim that the evidence established a causal link between unconscious conflict and conscious symptom experience. In the spider phobia study (section Previous Studies With Spider Phobia: Alpha Power Serves as an Inhibiting Brain Mechanism in Phobic Experience; Shevrin et al., 2010) we showed how alpha power functioned to inhibit responses to a subliminal phobic stimulus on different tasks (e.g., attention, detection, etc.). From a psychoanalytic standpoint, the important missing piece in the spider phobic study was the underlying unconscious conflict creating the necessary motivation for inhibition or repression of spider stimuli. It seemed clear that now we needed to show that alpha power served as the causal neural link between unconscious conflict and clinically based conscious symptom experience.

According to psychoanalytic conflict theory (Freud, 1955, 1957, 1959; Brenner, 1982), neurotic disturbances are the consequence of a conflict of motives operating largely unconsciously. Any effort to investigate this proposition must: (1) infer from appropriate clinical material that a particular conflict causes a specific neurotic disturbance, (2) demonstrate that only when the conflict is activated via subliminal presentation (so that it is active outside of the person's awareness) does it produce an inhibitory response in the conscious neurotic disturbance, (3) show that when the unconscious conflict stimuli are supraliminal (so that they are processed consciously) they will not produce these inhibitory effects specific to the conscious neurotic disturbance, (4) ascertain that when control conscious experiences other than the specific neurotic disturbance are involved there will be no evidence of an unconscious conflict inhibitory effect either subliminally or supraliminally. To summarize: The relevant unconscious conflict specific to the neurotic disturbance has its inhibitory effect on the neurotic disturbance only when the unconscious conflict is activated subliminally, and only with respect to that neurotic disturbance (i.e., the conscious symptom stimuli). In short, the inhibition is unconscious and specific to the particular neurotic disturbance.

But why should the inhibitory repressive process triggered by the activation of unconscious conflict also lead to inhibition related to the conscious symptom experience? In the current experiment, the critical condition involves initially subliminally presenting words related to participants' unconscious conflict, rapidly followed by words related to their conscious symptom experience. These conscious symptom words describe the aspects of the social situation that are most anxiety provoking and fearful to the participant, as well as the physiological signs of anxiety the participant experiences in the uncomfortable social situation. In effect, the presentation of these words places the participant consciously into the dreaded socially feared situation. But—and this is critical—these social situations are dreaded precisely because they themselves also resemble the participant's unconscious conflict. If they did not, participants would not exhibit symptoms in response to such situations. This fact highlights a fundamental point—that conscious symptoms (taken together with the situations that trigger them) are related to and interconnected with the underlying unconscious conflict. The participant experiences social situations as if they contain an aspect of the unconscious conflict, although the participant does not realize that their unconscious conflict is influencing their conscious experience. For example, if the participant's unconscious conflict revolved around his relationship with his father (as suggested by example in Supplement 3), then the participant will experience certain social interactions in the same framework as he experienced his conflictual interactions with the father. Consciously, it becomes a struggle between enjoying the social occasion and tolerating increasing anxiety generated by its unconscious significance. Thus, from a psychodynamic perspective, repression must be directed from the activated unconscious conflict toward the social situation, or the most (unconsciously) conflictual aspects of it. Accordingly, attempts to inhibit and repress activation of the unconscious conflict should predict attempts to inhibit and repress responses to the conscious symptom stimuli. Therefore, we would expect to find a positive correlation between inhibition of unconscious conflict, and inhibition of the following conscious symptom reminders. By contrast, purely cognitive accounts, which lack any concept of unconscious conflict, would predict no relationship at all between the two kinds of stimuli.

The novel methodological innovation in this new study was to show that inferences drawn from entirely qualitative psychoanalytic clinical material can be tested by objectively measurable brain processes so that what is ultimately demonstrated is an underlying commonality of function between psychodynamic and brain processes.

The major change from the original experimental design of the first social phobia study was to shift to a priming model in which the unconscious conflict stimuli preceded the conscious symptom stimuli. A priming model allowed us to empirically assess the effects of the unconscious conflict stimuli on the following conscious symptom stimuli, and to scrupulously control for any potential confounding influences. In all other respects the design of this new study, presented in detail below, was exactly the same as of our first study, described in Shevrin et al. (1992).

## Methods and materials

### Participants

Ten subjects (7 women, 3 men) who met DSM IV-R criteria for social phobia were selected. The subjects provided written informed consent prior to participation in the study. The study was approved by the University of Michigan Medical School Institutional Review Board (IRBMED) Ethics Committee.

### Procedure

After the participants had been selected, 3–4 hour taped diagnostic interviews were obtained from each participant and transcribed for examination and word selection [see Shevrin et al. (1996, 59–90) for details; Chapters 9, 10, 11 for detailed case studies]. This procedure yielded the Unconscious Conflict (UC) and Conscious Symptom (CS) primes, selected individually for each participant. Conscious Symptom (CS) word targets were also drawn from these interviews, and when such words appeared both as primes and targets they were always non-identical. A set of Osgood Negative Valence (ON) words, selected uniquely for each participant, served as the control (Osgood et al., 1975). For each of the word groups (UC, CS, and ON) judges selected seven words for each participant. The three word groups were equated for frequency, length, and part of speech (nouns and verbs). In Supplement 3, we present a sample set of words for each of the three categories of stimuli for one subject.

The selected words were presented tachistoscopically to the participants. The key experimental condition was subliminal unconscious conflict primes followed by supraliminal conscious symptom targets. This critical condition was embedded in a Prime Type (unconscious conflict vs. conscious symptom) × Target Type (conscious symptom vs. Osgood negative valence) × Prime Duration (subliminal vs. supraliminal) 2 × 2 × 2 within-participants factorial design. Every presentation consisted of two words, the prime and the target, presented 1000 ms apart. All primes were presented subliminally as well as supraliminally, whereas the targets were always supraliminal. Each prime word was paired with every target word for a total of 196 subliminal and 196 supraliminal trials (e.g., unconscious word prime 1 was paired with all 14 CS and ON targets). All subliminal prime trials were presented first, followed by all supraliminal trials. Otherwise, prime–target pairs were randomized. Participants were simply instructed that at a prearranged signal they should fixate on a dot in the center of a blank field and not blink or move their eyes until signaled that the trial was complete.

The subliminal stimuli were presented for 1 ms duration, 10 ft/lamb.; supraliminal duration was set at 30 ms, 10 ft/lamb. These durations are much briefer than in typical subliminal studies. We used a Gerbrand Model 3-field Dodge-type tachistoscope, which is capable of much faster durations than a computer that requires backward masking to achieve subliminal presentations. Ambient light was set at 10 ft./lamb., matching the luminance of the stimuli [see Shevrin et al. (1996) for successful application of these settings].

### Measures

EEGs were recorded from 10 electrodes (SCR, OB muscle, Corrugator muscle, F3, F4, CzPz, P3, P4, Oz, EOG) with reference to linked ears (A1 + A2). A 10–20 electrode placement system was used for application of electrodes. Silver-silver chloride electrodes were used with impedance kept below 5 K Ohms. Originally data was collected at 500 samples per second and then down sampled to half at 250 samples per second for 2 s and 400 ms. A total of 600 bins or 2400 ms of data were collected. Grass model 8 EEG machine was used to amplify EEGs and a hardware filter of 0.3–125 Hz was used. LabView on a Macintosh computer was used to digitize EEGs. Digitized data were then transferred to a LINUX PC for further analysis. MATLAB was used for doing FFT and Alpha band power calculations. Alpha power (8–13 Hz), calculated via the Fourier transform, was determined for the subliminal and supraliminal prime (1000 ms) and target (1000 ms) epochs post-stimulus for each trial, and then averaged within-condition (e.g., subliminal UC primes when followed by CS targets, etc.). For each participant there were a total of 49 trials averaged for each prime and target category. The experimenter visually inspected EEG recordings online for artifacts resulting from eye movement, muscle movement and 60 Hz. Trials containing such artifacts were rejected online. The stimuli were then repeated in the next trial and EEG data was re-recorded. Because we did not initiate trials until participants indicated their readiness and the ongoing EEG was artifact-free, very few trials (1–3%) needed to be repeated, and the proportions of such trials did not differ between conditions. Because the alpha power distribution was notably skewed across participants, alpha power was log-transformed before further analysis.

The measure of alpha power we used was absolute alpha power. We did not divide prime or target alpha by a baseline so the units in the figures simply reflect alpha power for both the *x*- and *y*-axes. While some researchers divide post-stimulus alpha by a preceding baseline, others often simply analyze unmodified post-stimulus alpha power. Both methods are equally valid. For example, Cooper et al. (2003) and Kelly et al. (2006), cited as prior research for our own, found increases in absolute alpha power to play inhibitory roles.

### Data analysis

Our main interest was in examining the influence of prime alpha power on target alpha power. To this end, we analyzed prime influences using a regression approach in which alpha power for the two primes (UC and CS) were predictors, and target alpha power was the dependent (criterion) variable. The regression approach allows estimation and comparison of the unique (i.e., uncorrelated) abilities of the two prime types to predict target alpha. In contrast, in the more commonly used difference score approach (e.g., UC—CS primes) to examining prime-target relationships, it is not possible to examine the contributions of the UC vs. CS primes independently, thus leaving it ambiguous as to which of the two factors caused a positive result. Although not presented here, the main difference results from these data largely converged with the regression results presented below.

One might also maintain that the main analysis, rather than regression, should have been a comparison of mean effects in an ANOVA. In many situations analyzing the means is helpful. Here, however, such analyses are of secondary interest, because we are critically interested in the *relationship* between prime and target alpha (i.e., the predictive/causal relationships between the two). Further, correlations as in regressions are independent of means and mean differences. For example, mean 1 could be larger than mean 2, but this says nothing about whether scores in condition 1 are correlated with scores in condition 2. We have previously documented in our review (Snodgrass et al., 2004a), extremely subliminal conditions (i.e., wherein detection *d*′ = 0) frequently yield exclusively *bidirectional* effects—that is, effects driven by individual differences but with no main (i.e., mean) effects. This is exactly the case with our data: An ANOVA on our data produced no mean effects, while the regression analysis was strikingly productive. For a comprehensive report of the data, we present means for all prime and target categories, as well as an ANOVA analysis in Supplement 4.

Because the subliminal vs. supraliminal prime duration manipulation is of great interest, we wished to analyze these conditions separately, so that any differences between subliminal vs. supraliminal effects would emerge. Regarding reporting R-square, often cited in regression analyses, in our case this would be uninformative or even confusing, for several reasons. First, we are only interested in possible differences in effects associated with UC vs. CS prime types (i.e., in regression language, their unique contributions to predicting target alpha). Such differences are captured precisely by the (unique) partial correlations currently reported in the text. In contrast, overall R-square reflects not only the predictors' unique contributions, but also any predictive contribution made by the predictors' shared (common) variance—and this variance is not of interest here. The current focus on unique prime type contributions to predicting target alpha removes these uninformative effects.

Our core hypothesis was that the degree of alpha power-related (i.e., inhibitory/repressive) brain activity in the UC primes should predict increased alpha power in the CS targets (i.e., signaling increased inhibition/avoidance), but not for unrelated ON targets. In keeping with our prior results from the first social phobic study (section The Initial Social Phobia Study: Establishing Clinical and Brain Evidence for Unconscious Conflict; Shevrin et al., 1992, 1996), this effect should manifest with UC subliminal but not UC supraliminal primes. In parallel fashion, any CS prime influences on CS or ON targets should manifest more strongly supraliminally than subliminally, an expectation based on the first social phobic study in which CS effects were only supraliminal (section The Initial Social Phobia Study: Establishing Clinical and Brain Evidence for Unconscious Conflict; Shevrin et al., 1992, 1996).

## Results

Four separate regressions were performed: (1) Subliminal primes (UC, CS) with CS target; (2) Supraliminal primes (UC, CS) with CS target; (3) Subliminal primes (UC, CS) with ON target; and (4) Supraliminal primes (UC, CS) with ON target. We began by averaging across all six electrodes (F3, F4, CzPz, P3, P4, Oz).

### Unconscious conflict primes (UC): subliminal and supraliminal results

**Figure 1:**
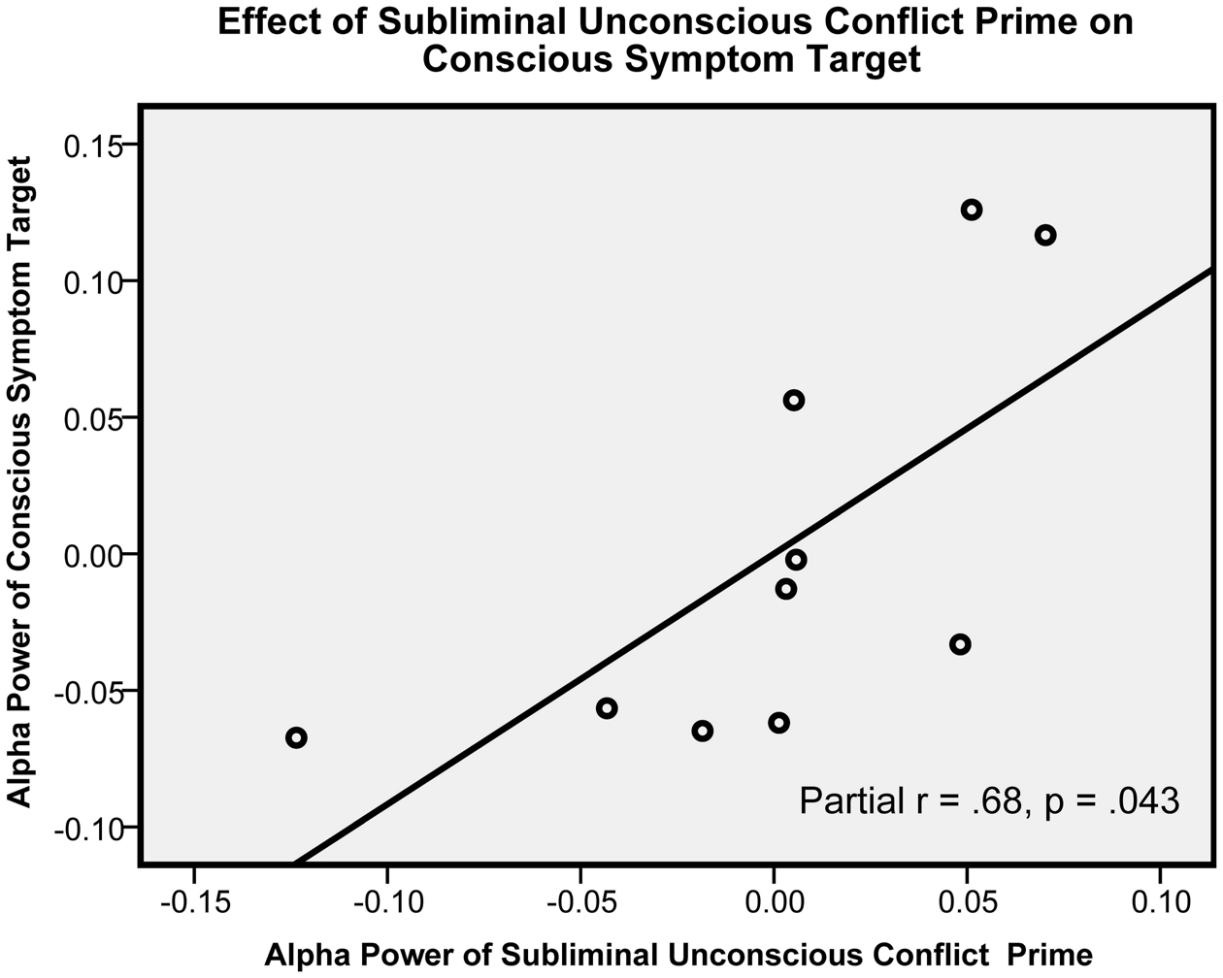
**This figure shows the key hypothesized relationship (partial r) between subliminal unconscious conflict prime alpha and the following conscious symptom target alpha.** Alpha power units are in squared microvolts, log-transformed. This graph includes measures averaged across all electrodes.

Subliminal UC prime alpha as hypothesized positively predicted CS target alpha, [*t*_(7)_ = 2.47, *p* = 0.043, β = 1.08, partial *r* = 0.68]. (The scatter plot showing this central hypothesized relationship is presented in Figure 1.) By contrast, subliminal UC prime alpha had no effect on ON control target alpha [*t*_(7)_ = 0.79, *p* = 0.45, β = 0.36, partial *r* = 0.29]. (The scatter showing an absence of an effect for this control condition is presented in Figure 3). When UC primes were supraliminal they no longer predicted CS target alpha: [*t*_(7)_ = 0.74, *p* = 0.49, β = 0.28, partial *r* = 0.27], and had no effect on control ON targets [*t*_(7)_ = −0.81, *p* = 0.45, β = −0.44, partial *r* = −0.29]. Thus, only when the UC primes were subliminal, and only when they preceded the CS targets did they produce an enhanced alpha effect.

### Conscious symptom primes (CS): subliminal and supraliminal results

**Figure 2:**
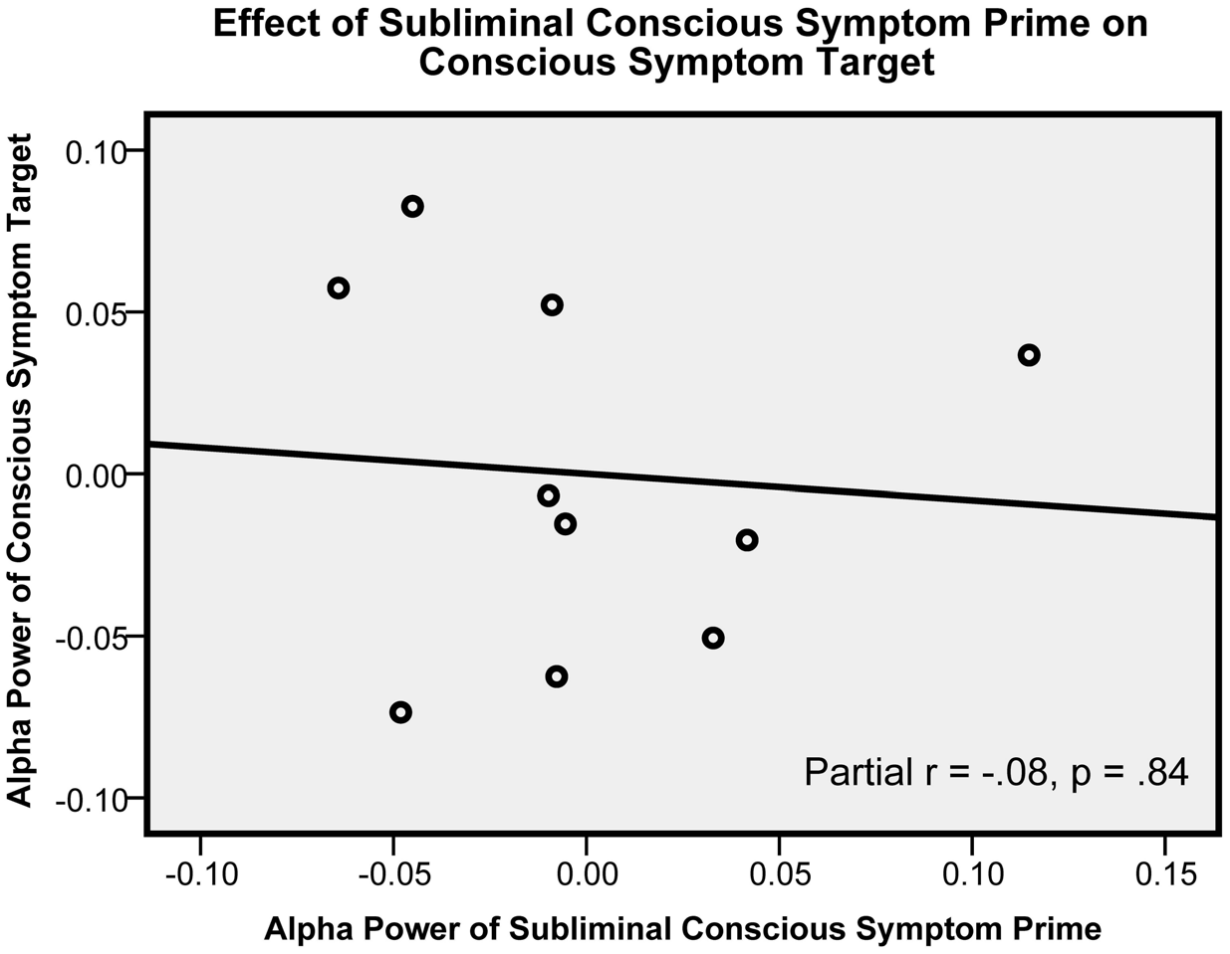
**This figure shows a control condition, in which the relationship present in Figure 1 disappears.** In this figure, subliminal conscious symptom primes were presented (instead of the subliminal unconscious conflict primes in Figure 1). The graph shows the partial *r* between subliminal conscious symptoms prime alpha and conscious symptoms target alpha. Alpha power units are in squared microvolts, log-transformed. This graph includes measures averaged across all electrodes.

Subliminal CS primes, unlike subliminal UC primes, did not predict CS symptom alpha [*t*_(7)_ = −0.21, *p* = 0.84, β = −0.09, partial *r* = −0.08]. (The lack of a relationship in this control condition is shown in the scatter plot in Figure 2.) The subliminal CS primes also did not predict control ON target alpha [*t*_(7)_ = 1.38, *p* = 0.21, β = 0.63, partial *r* = 0.46]. Supraliminal CS primes came close to positively predicting CS target alpha: [*t*_(7)_ = 1.81, *p* = 0.11, β = 0.70, partial *r* = 0.56], and did positively predict ON target alpha [*t*_(7)_ = 2.62, *p* = 0.034, β = 1.42, partial *r* = 0.70]. Finally, given the similar results for supraliminal CS primes with both CS and ON targets, we also pooled both targets, yielding an overall near-significant result; [*t*_(7)_ = 2.15, *p* = 0.069, β = 1.11, partial *r* = 0.63].

**Figure 3:**
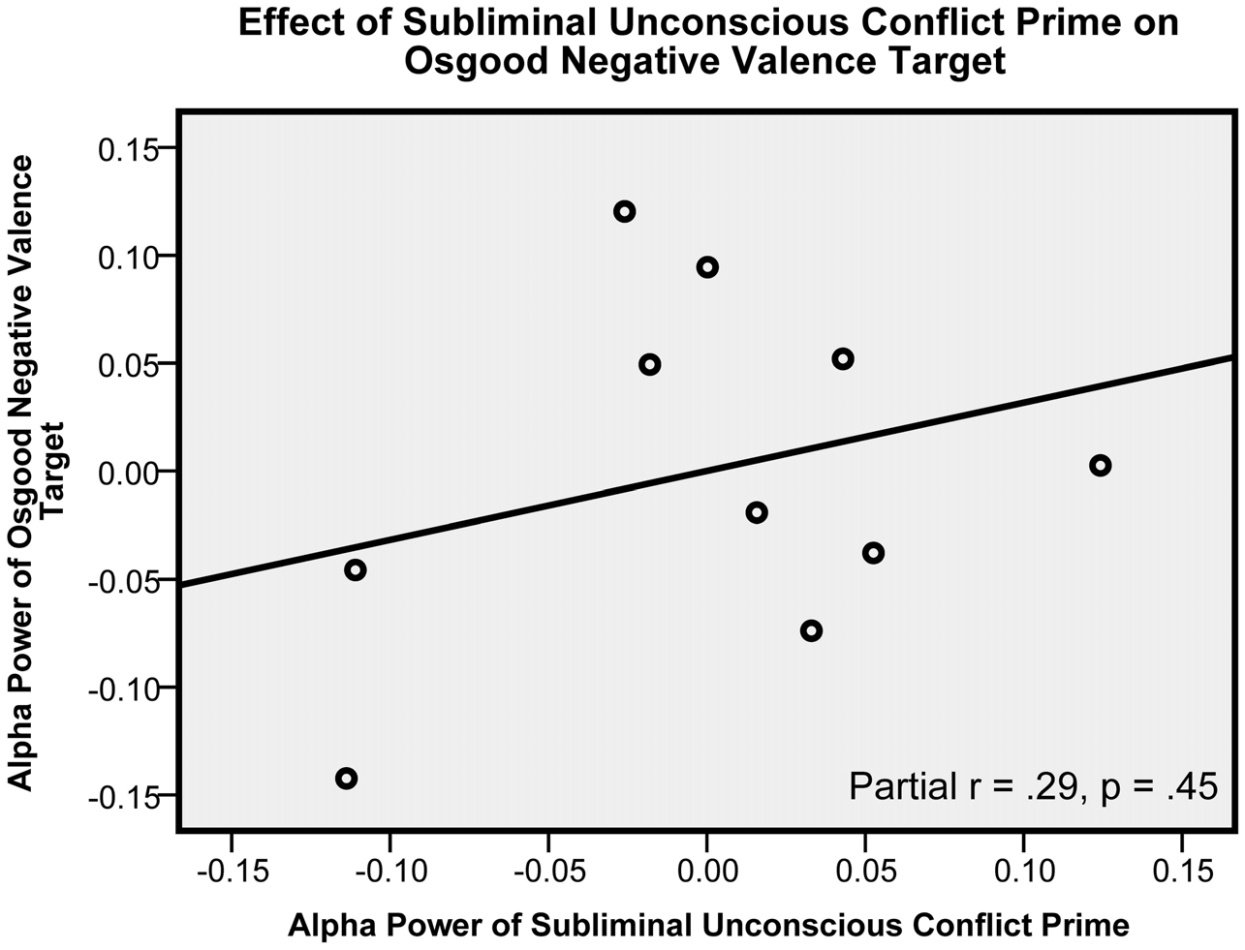
**This figure shows that the relationship presented in Figure 1 is absent in another control condition.** Here, the conscious symptoms targets (as presented in Figure 1) are replaced with the control Osgood negative valence target. The graph shows the partial *r* between subliminal unconscious conflict prime alpha and Osgood negative valence target alpha. Alpha power units are in squared microvolts, log-transformed. This graph includes measures averaged across all electrodes.

### *Post-hoc* findings

Upon examining parallel analyses for individual electrodes, we noticed an apparent frontal (F3, F4, CzPz) vs. parietal (P3, P4, Oz) pattern. We then averaged across these electrode subgroups. For economy of exposition we focus on the main results; other findings never approached significance and showed no frontal vs. parietal differences. These analyses suggested that the critical subliminal UC/CS finding was strongly present parietally: UC prime alpha [*t*_(7)_ = 3.95, *p* = 0.006, β = 1.37, partial *r* = 0.81], but non-significant frontally: UC prime alpha [*t*_(7)_ = 1.16, *p* = 0.28, β = 0.57, partial *r* = 0.40]. Conversely, the supraliminal CS prime findings were stronger frontally [CS target: *t*_(7)_ = 2.33, *p* = 0.05, β = 0.65, partial *r* = 0.66; ON target: *t*_(7)_ = 3.67, *p* = 0.008, β = 1.27, partial *r* = 0.81], but non-significant parietally [CS target: *t*_(7)_ = 1.43, *p* = 0.20, β = 0.69, partial *r* = 0.48; ON target: *t*_(7)_ = 1.27, *p* = 0.24, β = 1.01, partial *r* = 0.43]. Again we note the inverse reciprocal relationships between unconscious conflict and conscious symptom results as a function of category and duration, paralleling findings from the first social phobic study (Shevrin et al., 1992, 1996).

### Bootstrap approach regressions

While the above findings confirmed our key hypotheses, one might worry that regression methods with small samples such as ours could yield unreliable results. Bootstrap methods, which repeatedly reanalyze the sample data using random resamples, are a useful tool to check this concern because they non-parametrically estimate the sampling distribution using the actual sample data rather than relying on standard assumptions (Fox, 2008). Here, we reran our primary regression analyses, now incorporating related bootstrapping techniques. For all electrodes combined, the critical subliminal UC prime/CS target effect actually improved slightly (*p* = 0.018), while the subliminal CS prime/CS target effect remained non-significant (*p* = 0.79), as did both UC prime controls (subliminal UC prime/ON target, supraliminal UC prime/CS target), *p*s > 0.35. Further, the relevant univariate distributions and regression diagnostics indicated no outliers with these key effects, suggesting these findings were not distorted by this potential problem. Finally, the non-predicted supra CS prime/CS target and supra CS prime/ON target effects also improved slightly (*p*s = 0.084 and 0.009, respectively). Overall, then, applying bootstrapping to the critical effects suggested they were reasonably stable.

## Discussion

The pattern of results supported our main hypothesis for unconscious conflict. Only when the unconscious conflict primes were subliminal did they significantly predict conscious symptom target alpha power. The alpha effect was not present: (1) when the unconscious conflict primes were supraliminal, (2) when the conscious symptom stimuli were the subliminal primes (3) when the control Osgood Negative words were the supraliminal target stimuli. In short, there was only one condition in which the unconscious conflict primes were associated with enhanced alpha power: when the subliminal unconscious conflict primes were followed by supraliminal conscious symptom targets.

These findings strengthen the interpretation of a cause and effect relationship between unconscious conflict and conscious symptom experience. Of particular importance to supporting our hypotheses, we did not simply obtain some single, isolated finding consistent with this hypothesis. Rather, based on coherent, interrelated psychoanalytic theory relevant to our hypothesis, we predicted—and obtained—an interrelated *pattern* of findings, including not only specifying where we *should* obtain the predicted result (i.e., with subliminal UC primes and CS targets), but also where we *should not* obtain the result (i.e., with ON targets or with supraliminal UC primes). Notably, we obtained this entire pattern, strengthening the likelihood that the key results are genuine—and meeting specific recommendations by Grunbaum regarding testing this fundamental psychoanalytic causal hypothesis.

The one seemingly anomalous finding, not in itself central to our hypotheses, was the alpha power effect found for the supraliminal CS primes and the ON target alpha power. Since these results were not predicted, they may possibly represent a false positive finding. Nevertheless, one might wonder that if any inhibitory effects can occur with CS primes whether that challenges the specificity of the relationship between UC primes and repression. We deal in some detail with this troublesome issue below, calling attention in particular to the findings that CS prime inhibition never occurs subliminally, it is not correlated with repression in the initial social phobia study, and it does not cleanly discriminate between conscious symptom experience and the ON control targets [the near significant conscious symptom result (*p* = 0.11)], suggesting that the inhibition is directed at negative valence words rather than specifically directed at the CS primes.

From this pattern of converging experimental and control results we are in a position to infer that only the unconscious conflict stimuli selected *a priori* by psychoanalysts from clinical data *causally link clinical inferences based on psychological meaning (unconscious conflict over emotionally incompatible desires) with brain processes (patterns of electrophysiological inhibition).* If so, then to our knowledge this is the first *psychophysiological* evidence for Freud's unconscious conflict theory of psychopathology. Repression emerges as a function of these inhibitory patterns, as it does in the patterns of psychological avoidance and choice. From this standpoint, repression is not a neural or psychological “force,” but a series of unconscious decisions creating a pattern of interactions with oneself and the world. That was what we found with the pattern of interactions for the unconscious conflict primes in the current study in which inhibition occurs subliminally, but not supraliminally.

Could alternative cognitive explanations account for some of the effects described in our results? The simple answer is that semantic priming would not predict any relationship at all between the unconscious conflict primes and the conscious symptom targets because the unconscious conflict primes do not constitute a semantic category at all, let alone one with any relation to the conscious symptom category. Indeed, as mentioned in the text, the participants in our earlier social phobia study themselves did not regard the unconscious conflict primes as a category when asked to consciously categorize the stimuli post-experimentally. On the other hand semantic priming accounts could explain the results involving the conscious symptom primes and conscious symptom targets, as these do constitute standard “related” categories. This is perfectly fine with us; we never thought such accounts would not apply to purely conscious symptom-related effects. Similar reasoning applies to emotion-attention accounts; like semantic priming, they would predict no relationship between UC primes and CS targets (because the UC primes do not comprise a standard emotional category), but could account for relationships between CS primes and CS targets (which do). Furthermore, attentional blink phenomena require much shorter prime-target SOAs (c. 200–300 ms) than ours (c. 1000 ms), so this mechanism could not account for our results.

What is it that needs to be explained and further understood about the phobic experience and how would our findings based on psychoanalytic concepts advance that understanding? It is clear that the phobic experience is one of fear, anxiety, avoidance, at times revulsion and disgust. Most important is the general observation that the phobic person is aware that these reactions are not substantiated by the nature of the actual phobic object or situation. Moreover, there is no agreed upon explanation on how a phobia develops or is sustained. For the psychoanalyst there might be an answer to be found to these questions when the role of unconscious conflict is taken into account. Perhaps surprisingly, we learn that it is not the phobic object or situation taken in its literal significance that is the source of the phobic experience amply reflected in the conscious symptom accounts. Instead, it is the way these accounts are related in significance to unconscious conflict, so that the conscious symptom experience falls prey to the same repressive influences as are present in the unconscious conflict and are reflected in our key findings. The conscious phobic experience is transformed into a stage on which invisible actors reenact the unconscious conflict scenario, out of keeping with what is happening visibly. Thus, the repression triggered by the activation of the unconscious conflict, should in a similar manner be directed toward the conscious symptom experience. Our results support this understanding with new evidence drawing on brain based inhibitory processes, as well as clinically based accounts of unconscious conflict. However, the influence of unconscious conflict on conscious symptom formation, and the role of repression and inhibition in this process presents a complex system which may unfold differently in different situations, such as different psychopathology or different experimental measures. The exact mechanism of this complex relationship is for future research to determine. But our findings tell us that, beyond the immediate largely conscious avoidant reactions, there are important new parameters to be take into account, namely unconscious conflict and repression.

Two findings require further examination: (1) for the unconscious conflict primes to have an alpha power effect on the conscious symptom experience they must first be subliminal, (2) whereas the conscious symptom primes exercise alpha power effects only supraliminally.

### The subliminal condition and alpha power effects at the objective detection threshold (ODT)

At the beginning of the senior author's program of research on unconscious processes, he decided to present stimuli at the fastest duration possible with equipment available at the time. The tachistoscope was the equipment of choice because it could deliver reliably (checked by oscilloscope) a 1ms duration at moderate luminance (10 ft/candles). Under these conditions stimulus detection was at chance (50%). In a series of studies (Shevrin, 1973) replicable subliminal effects were obtained under these conditions.

Snodgrass in a series of subsequent studies (Snodgrass et al., 1993a,b, 2004a,b) combined a signal detection theory approach with Shevrin's earlier psychophysical method, making it possible to go beyond the limitations of classical psychophysics and assess accuracy separately from bias. This made it possible to obtain an accurate measure of the objective detection threshold (ODT) free of criterion bias at which detection (*d*′) was not different from zero. Subsequent research revealed that at the ODT different things happen than at the supraliminal threshold. For example, in a series of replicated studies (Snodgrass et al., 1993a,b, 2004a,b), Snodgrass discovered inhibitory processes at the ODT that were a function of individual differences, findings quite germane to our current study. These effects were based on standard cognitive tasks (e.g., word choices). At the ODT different kinds of thought processes might be occurring. This was borne out in another study on language processing in which palindrome, or reverse word priming, was found at the ODT, but not in the supraliminal condition where only standard forward priming occurred (Villa et al., 2006a,b). The ODT may not only be the purest unconscious state our current methods can produce, but may also constitute a qualitatively different psychophysiological state susceptible to unconscious conflict activation. (It is also possible that similar findings might occur at other subliminal thresholds such as the subjective threshold at which *d*′ > 0, but participants aver that they do not see the stimulus, hence subjective (Snodgrass et al., 2004a,b). This remains for future research to determine). These ODT findings raise the possibility that when the unconscious conflict primes are processed at the ODT they become sensitive to individual differences, among them, the inhibitory and repressive processes we have found that do not happen when the same words are processed supraliminally.

### The supraliminal condition and the conscious symptom experience

If the reasoning offered above for the unconscious conflict primes at the ODT is correct, why doesn't it also work for the conscious symptom primes when they are subliminal, but appear to work only when they are supraliminal? Here two findings from the initial social phobic study (section The Initial Social Phobia Study: Establishing Clinical and Brain Evidence for Unconscious Conflict) are pertinent. First, the repressiveness personality measure predicted t-f subliminal vs. supraliminal classification for the unconscious conflict primes only. This relationship was not found for the conscious symptom stimuli. Moreover, supraliminally the unconscious conflict primes were not significantly grouped together either by the t-f feature analysis or by participants' own category groupings. They simply did not form a category supraliminally (Shevrin et al., 1992, 1996). This was further confirmed by Kushwaha et al.'s (1992) information flow analyses, which showed that the unconscious conflict category showed more information flow subliminally than supraliminally, and more than control pseudo-category analyses. Thus, the unconscious conflict primes were a meaningful category only subliminally in the first study (section The Initial Social Phobia Study: Establishing Clinical and Brain Evidence for Unconscious Conflict), and it was only this category that had a significant subliminal alpha power inhibitory effect on the supraliminal conscious symptom targets, in the new, or second social phobic study (section The New Investigation: Establishing a Repressive Causal Link Between Unconscious Conflict and Conscious Symptom Experience). By contrast, participants in the initial study (section The Initial Social Phobia Study: Establishing Clinical and Brain Evidence for Unconscious Conflict) had no difficulty in treating the supraliminal conscious symptom primes as a category expressing what the participant had previously described as disturbing about the phobic experience. None of this could be said about the supraliminal unconscious conflict primes (Shevrin et al., 1992, 1996).

On these grounds it seems reasonable to suppose that the supraliminal conscious symptom words were overtly disturbing to the participants because they brought back the conscious social phobic experience itself. And when at the same time participants in the second study (section The New Investigation: Establishing a Repressive Causal Link Between Unconscious Conflict and Conscious Symptom Experience) were exposed supraliminally to other quite negative stimuli (other conscious symptom words or Osgood Negative words), participants would be disposed to respond with efforts at inhibition or avoidance, now completely conscious.

When we compare the *supraliminal conscious symptom prime condition* with the *subliminal unconscious conflict prime condition*, in both of which evidence for inhibition appears, differences are found that inform us of the difference between conscious and unconscious inhibition. For example, there is no evidence that the inhibition following the supraliminal conscious symptom prime condition has any of the characteristics of repression. In contrast, multiple sources of evidence point to an association between the subliminal unconscious conflict prime condition and repression. The unconscious conflict prime condition showed a relationship to the repression personality measure in the first study (section The Initial Social Phobia Study: Establishing Clinical and Brain Evidence for Unconscious Conflict; cf. Shevrin et al., 1992, 1996), as well as to an independent projective behavioral measure of repression, with both of these indexes of repression being highly correlated (Caine and Hawkins, 1963; Caine and Hope, 1967; Ludolph, 1981; Shevrin et al., 2002a,b). But at the heart of the matter is the second study's central finding that only the subliminal unconscious conflict primes activate the unconscious conflict; while the supraliminal conscious symptom prime condition activates the conscious phobic experience. This difference between what the participant is aware of fearing consciously and seeks to avoid consciously and what constitutes repressed unconscious knowledge related to these conscious fears lies at the heart of the psychoanalytic conception of psychopathology.

## Limitations

Although a substantial amount of prior work (cited above) supports the inhibition interpretation that we provide for alpha power, some (e.g., Palva and Palva, 2007) have suggested other functions of alpha. However, we note their position is based on phase-locked alpha, not non-phase-locked alpha as in our research and in the alpha/inhibition literature generally (e.g., Kelly et al., 2006). Also, Palva and Palva's alternative interpretation is specifically linked to supraliminal stimuli (see their p. 157, box), and is hence likely not applicable here in any case.

It also might have been useful to have here included a behavioral measure as an independent indicator of inhibition. We note, however, that our previous spider phobia study (Shevrin et al., 2010) suggested that greater alpha power was indeed associated with inhibited performance on a behavioral signal detection task. Nonetheless, our and others' future work would benefit from routinely including behavioral measures of inhibition. We also note that our stimulus materials (especially UC words), while balanced on many factors (frequency, length etc.), could not be balanced for all possibly relevant dimensions (e.g., arousal). This is a necessary limitation due to their completely individualized nature, a unique feature of this study perhaps essential to investigating the impact of intrinsically idiographic UC (and to a lesser extent, CS) stimuli—a fundamental goal of the current line of research.

Finally, our sample size is small, which raises potential statistical bias/instability issues with regression approaches such as ours. The bootstrap analyses, however, suggested our regression results were reasonably stable, and there was no indication of potentially distorting outliers. Nonetheless, because this is a small-sample single study, further replication with larger samples is needed to more firmly establish our conclusions. We plan such a larger-scale replication, which would additionally include other valuable data-analytic approaches such as single-trial analyses and robust regression methods, as well as a behavioral measure of inhibition.

## Future directions

One future direction would take us to applied clinical research. We would expect that following successful psychodynamic, conflict oriented treatment, the same unconscious conflict prime words presented after treatment would no longer have an enhanced inhibitory effect on conscious symptom targets. It would no longer be needed. Another direction might take us into the curious inverse reciprocal relationship between conscious and unconscious processing as a function of threshold. We have suggested that this inverse reciprocal relationship can be understood as determining what is limited to conscious processing as contrasted with unconscious processing. From this standpoint the Snodgrass discovery of the ODT may open the door to investigations of the complex relationships between conscious and unconscious processing in normal and abnormal states.

Lastly, our findings may offer an opening for studying the neurophysiology of repression. The *ad hoc* results of the second study suggest that the repressive effect of the subliminal unconscious conflict primes is more closely associated with parietal alpha, while the alpha power effect of the supraliminal conscious symptom primes is more closely associated with frontal alpha. Also it seems that repression emerges as the outcome of complex interacting decisions and choices rather than as a punctiform cause acting at one particular time.

## Conclusion

We set out to seek evidence for a cause and effect relationship between unconscious conflict alpha power and conscious symptom experience. Our findings supported our hypothesis. Only when the unconscious conflict primes were subliminal did they have an inhibitory effect on the processing of conscious symptom targets, and this relationship was physiologically instantiated through inhibitory alpha power activity. The study also yielded unexpected findings concerning the effects of supraliminal conscious symptom primes. In both the initial and the new social phobic studies, the effects of conscious symptom primes and unconscious conflict primes appeared inversely related to each other. Specifically, in the new social phobic study, the unconscious conflict primes only produced inhibition when presented subliminally and only selectively for conscious symptom targets, while conscious symptom primes only produced inhibition when presented supraliminally, and did not discriminate between targets. We concluded that the supraliminal conscious symptom effects were due to conscious re-experiencing of the phobia with attendant efforts at conscious inhibition and avoidance, as occur in many cognitive phobia experiments. Only when subliminal unconscious conflict primes enter the picture is another level of meaning involved that engages repression. We can thus distinguish between conscious inhibition and unconscious repression, only the latter involving unconscious conflict.

## Conflict of interest statement

The authors declare that the research was conducted in the absence of any commercial or financial relationships that could be construed as a potential conflict of interest.
