# Supplementary material for: Subliminal unconscious conflict alpha power inhibits supraliminal conscious symptom experience
Source: Front Hum Neurosci. 2013 Sep 5;7:544. doi: 10.3389/fnhum.2013.00544 (PMC3763585; doi:10.3389/fnhum.2013.00544)
Supplement: Supplementary file 1 [file Presentation_1.PDF]

Supplementary Online Information

Supplement 1:

Here we provide additional technical information regarding the time-frequency distribution (TFD) and the information flow technique.

Time Frequency Distribution: A time domain signal is the raw signal collected from a source such as the ERPs collected from participants from this initial social phobia experiment. It depicts the amplitude of ERPs varying over time in 2 dimensions (time and frequency). Fast Fourier Transform (FFT) converts the time domain ERP into frequency domain signals, which display power at each of the possible frequencies and times in the ERP signal. For a fast, time varying signal such as EEG and ERP, FFT cannot provide frequency and power for the very short time bins in EEG/ERP signals. The time-frequency distribution (TFD) handles this by calculating and depicting power for each possible time and frequency bin in three dimensions (time, frequency and power). The Standard TFD has a limitation of having cross terms-between two frequencies, which muddles the TFD. Our improved Reduced Interference Distribution (RID) is a type of TFD which reduces the interference or cross terms. RID was originally used to analyze clinical ERP data. Several time and frequency features were selected in such a way so that they can differentiate best between the two critical (UC and CS) categories (Shevrin et al., 1992, 1996).

Information flow is category related mutual information calculated between two channels [e.g. left and right parietal (P3 and P4) of ERP data] for all combinations of time bins (e.g. between time bin 1 of P3 channel and time bin 2, 3, 4... up to last time bin of P4 channel). The *mutual information*, also known as transinformation, of two channels of EEG is a quantity that measures the mutual dependence of the two channels of EEG. It measures how much one channel depends upon the second channel. It is a dimensionless unit, most commonly expressed in "bits". High information flow, meaning high mutual information between two channels, indicates a large reduction in uncertainty. Low information flow, meaning low mutual information between two channels, indicates low reduction in uncertainty. Finally, zero information flow, meaning no mutual information between two random channels, indicates the two channels are independent. Superior to standard techniques, our method indexes only category-related (not background) channel dependence--that is, mutual information between two channels (Kushwaha et al., 1992).

Supplement 2:

This poster abstract originally appeared in the proceedings of the Society of Biological Psychiatry 2010 Annual Meeting (Shevrin et al, 2010).

105. Evidence for Unconscious, Perceptual Avoidance in Phobic Fear

Howard Shevrin[1], Michael Snodgrass[1], James L. Abelson[1], Linda A. W. Brakel[1], Ramesh Kushwaha[1], Hedieh H. Briggs[1], Ariane Bazan[2]

[1]Psychiatry, University of Michigan, Ann Arbor, MI,
[2]Psychological and Education Sciences, University of Brussels, Brussels, Belgium

Background: Anxious patients show attentional bias reflecting automatic, rapid vigilance for threat that may contribute to symptom maintenance. Threat avoidance also characterizes anxious patients, but is thought to occur later as a secondary, conscious process, however, attentional inhibition can occur rapidly and unconsciously. Synchronized alpha event-related potentials (a-ERS) are thought to reflect attentional inhibition. The N100 ERP also reflects early attentional processes. We examined these measures in spider and snake phobics subliminally exposed to spider stimuli to explore possible early perceptual avoidance of threat cues.

Methods: Ten spider and 7 snake phobics were exposed to spider and control cues in a subliminal exposure paradigm with ERP and subjective response measures, followed by post-exposure signal detection testing to insure objective subliminality.

Results: N100 amplitude was enhanced for spider phobics responding to spider cues. A greater a-ERS effect was associated with diminished N100 amplitude and delayed latency. A greater a-ERS effect was also associated with below chance detection of spider cues, greater spider fear, and less reduction in fear after repeated exposures.

Conclusions: Alpha-ERS has been shown to reflect inhibition of attention to distracting, conscious, neutral stimuli. Our data suggest that a-ERS is also associated with rapid, unconscious inhibition of attention to salient emotional cues. The a-ERS, N100, fear and fear change data converge to suggest that avoidance of a phobic stimulus can occur unconsciously. Threat perception in phobics may reflect a balance between threat vigilance and threat avoidance, and both can occur very early in perceptual processing.

Supplement 3: example of stimuli words

**Unconscious Conflict**	**Conscious Symptom**	**Osgood Negative Valence**
mad at dad	cold calling	air pollution
paddle me	public talks	atomic bomb
favorite son	voice shaking	earthquake
cut it off	seminars	non-believers
trouble maker	upset stomach	crying
stubborn	stuttering	noise
grandpa	parties	poison

Supplement 4

Here we include an analysis of means, in addition to the primary regression approach reported in the main text. The analysis of means includes descriptive statistics and an ANOVA analysis of the data. The descriptive statistics include the means and standard deviations of alpha power in the eight target trial groups and eight prime trial groups collected in the study. All trials were grouped by prime type (UC or CS), prime duration (subliminal or supraliminal), and target type (CS or ON), generating eight groups of trials. Data were collected during the prime window as well as during the target window of each trial. The means and standard deviations for all sixteen groups are presented in Table 1 and Table 2 below:

Table 1. Mean and SD of Alpha Power in Prime Window for Eight Stimuli Groups

**Trial type**	**Mean Alpha Power**	**Standard Deviation**
CS subliminal prime, CS target	3.183	.442
CS subliminal prime, ON target	3.181	.435
UC subliminal prime, CS target	3.184	.466
UC subliminal prime, ON target	3.190	.453
CS supraliminal prime, CS target	3.169	.428
CS supraliminal prime, ON target	3.165	.412
UC supraliminal prime, CS target	3.143	.360
UC supraliminal prime, ON target	3.169	.425

Table 2. Means and SD of Alpha Power in Target Window for Eight Stimuli Groups

**Trial type**	**Mean Alpha Power**	**Standard Deviation**
CS subliminal prime, CS target	3.119	.394
CS subliminal prime, ON target	3.116	.377
UC subliminal prime, CS target	3.104	.400
UC subliminal prime, ON target	3.114	.427
CS supraliminal prime, CS target	3.126	.385
CS supraliminal prime, ON target	3.148	.395
UC supraliminal prime, CS target	3.131	.338
UC supraliminal prime, ON target	3.131	.346

Our main hypotheses for this study concerned a causative, predictive relationship between the prime and target. This relationship, measured through a regression approach in the main results section is statistically independent from any group differences between mean alpha power level in any of the prime and target trial groups. Nonetheless, we conducted an exploratory analysis to test for mean differences between alpha power in different target conditions. We conducted a (2*2*2) ANOVA, with the factors: prime type (UC or CS primes) * prime duration (subliminal or supraliminal) * target type (CS or ON targets). The interaction was not significant [$F(1, 9) = 1.119$, $p=.302$]. This provides another example, in addition to earlier work (Snodgrass et. al, 2004; Etkin, et al., 2004), of subliminal effects yielding significant correlational results in the absence of mean group differences.
